# On the Mechanical Properties and Uncertainties of Jute Yarns

**DOI:** 10.3390/ma10050450

**Published:** 2017-04-25

**Authors:** AMM Sharif Ullah, Sweety Shahinur, Hiroyuki Haniu

**Affiliations:** 1 Department of Mechanical Engineering, Kitami Institute of Technology, 165 Koen-cho, Kitami, Hokkaido 090-8507, Japan; harry@mail.kitami-it.ac.jp; 2 Graduate School of Engineering, Kitami Institute of Technology, 165 Koen-cho, Kitami, Hokkaido 090-8507, Japan; 170178sweety@gmail.com

**Keywords:** natural materials, jute, mechanical properties, uncertainty quantification, possibility

## Abstract

Products made from natural materials are eco-friendly. Therefore, it is important to supply product developers with reliable information regarding the properties of natural materials. In this study, we consider a widely used natural material called jute, which grows in Bangladesh, India, and China. We described the results of tensile tests on jute yarns, as well as the energy absorption patterns leading to yarn failure. We have also used statistical analyses and possibility distributions to quantify the uncertainty associated with the following properties of jute yarn: tensile strength, modulus of elasticity, and strain to failure. The uncertainty and energy absorption patterns of jute yarns were compared with those of jute fibers. We concluded that in order to ensure the reliability and durability of a product made from jute, it is good practice to examine the material properties of yarns rather than those of fibers.

## 1. Introduction

We inhabit both artificial and natural worlds. The artificial world is full of products, each of which has a life cycle (conceptualization, design, manufacturing, marketing, use, service, recycling, disposal, and landfilling). Producing a product and supporting its life cycle requires primary materials and energy. To obtain primary materials and energy, we need resources, which come from the natural world. This means that we influence the natural world by enriching our artificial world with products. Because both worlds must coexist, we must not overburden the natural world; simultaneously, we must fulfill the needs of our artificial world. This leads to a concept called sustainability, which deals with issues related to the coexistence of natural and artificial worlds [1,2]. Many strategic goals have been set to achieve sustainability. One of the goals is to reduce the CO_2_ footprint by half, by the year 2050 [3]. This requires the simultaneous attainment of efficiency in terms of materials, energy, and components/products [3,4,5,6,7]. Specifically, improving material efficiency means increasing the use of environmentally friendly materials, increasing material yields, making lightweight products, etc. [3,4,5,6,7]. Energy efficiency means deploying renewable energy sources, decreasing energy usage, etc. [6,7]. It has been found that material efficiency is more effective than energy efficiency in achieving the strategic goals of sustainability [3,4,5,6,7]. One of the options for achieving material efficiency is to increase the amount of usage of natural materials in various products as much as possible [8]. Similar to other engineering materials (e.g., metals and alloys, composites, technical ceramics, and polymers), we need to know the properties of natural materials before designing a product to ensure product functionality, quality, durability, and reliability [8]. Knowledge of material properties comes from experimental investigations (e.g., tensile tests, flexural tests, hardness tests, etc.). The properties of natural materials vary significantly, because the microscopic structures of a naturally growing material cannot be tightly controlled [9]. This causes variability in the underlying properties of a material. Therefore, understanding natural materials requires a clear understanding of the uncertainty associated with each relevant material property [10]. Otherwise, it is not possible to make design and manufacturing decisions that will ensure the functionality, quality, reliability, and durability of natural material-based products.

In this study, we considered a widely used natural material called jute, which grows mainly in Bangladesh, India, and China [11]. We described the results of tensile tests on jute yarns. We also quantified the uncertainty associated with some jute yarn material properties (as determined from tensile testing), and described the failure mechanisms of jute yarns, using an energy absorption approach. The objective of describing these parameters was to better understand the material properties of jute in terms of reuse potential (e.g., making design decisions to ensure the functionality, quality, durability, and reliability of jute products).

The remainder of this article is organized as follows. Section 2 describes the primary production of jute and relevant studies that have been done on jute and its composites. Section 3 describes the tensile tests performed to measure the mechanical properties of jute yarns. Section 4 describes the energy absorption pattern of jute yarns (and jute fiber) as a cause of their failure. Section 5 describes the uncertainty associated with mechanical properties of jute yarn (jute fiber, as well). Section 6 presents concluding remarks.

## 2. Jute and Related Studies

**Figure 1:**
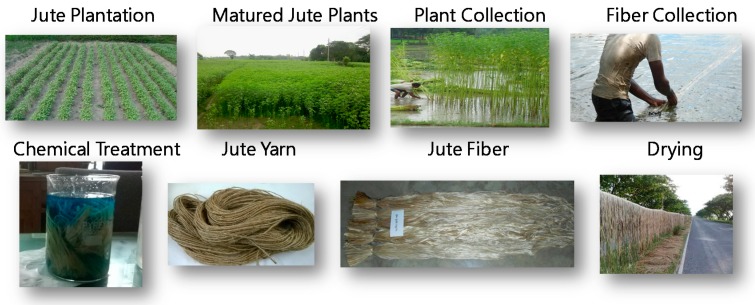
Producing jute fiber/yarn.

As mentioned above, jute is one of the most widely used natural materials [11], and is of interest for the development of eco-products [8]. Accordingly, numerous products are made from jute fibers, yarns, and composites. Such jute-based products as sacks, bags, corrugated sheets, carpets, shoes, sandals, and fabrics are now available in the market. The demands of these products are growing significantly, as they are eco-friendly products. Figure 1 shows a typical scenario of primary production of jute fibers and yarns. As seen from Figure 1, jute is grown in plantations, where it is collected after it matures. The plants are then typically soaked in water so that the fibers can be more easily collected. Other processes to collect the fibers without soaking may be used, if preferred. The jute fibers are then dried and marketed for further processing. Yarn is often produced from jute fibers. Sometimes, yarns are chemically treated to enhance their material properties.

Developing products using jute and other natural fibers requires an understanding of their material properties [8]. To understand the properties of jute-based products, two types of experiments have been performed. The first type deals with the characterization of composites, where jute and other natural/artificial fibers are used as reinforcing materials [12,13,14,15,16,17,18,19,20,21,22,23,24,25,26,27]. The main concern of these studies is to elucidate the efficacy of methods for improving the material properties of composites. The following issues have been studied: improving the dynamic mechanical properties of a jute composite [27], improving the adhesion between a matrix and fibers using chemically treated fibers [12,13], improving the performance of a composite by changing the weight percentages of fibers [13,14], improving the performance of a composite by changing the fiber length [15,16] and orientation [17,18], improving the performance of a composite through lamination [12,17,18,19,20], improving the performance of a composite by mixing jute fibers with other natural or artificial fibers [18,21,22,23,24,25,26], etc. Here, “improving performance” means improving the mechanical, thermal, environmental degradability, and durability properties of a composite.

The other type of experiments performed on natural materials (including jute) has aimed to determine the properties of raw or chemically treated fibers collected from various segments of the respective plants [28,29,30,31,32,33,34,35,36,37,38]. In particular, some studies have determined the properties of raw fiber [34,35,36]. There are other studies that have determined the properties of chemically treated fibers [37], as well as the fibers collected from various segments of jute plants [36]. The goal of such studies has been to gain scientific knowledge about the fiber itself, which can then be applied in designing jute or jute composite products. For example, see [8] to understand how the material properties of jute fibers have been used to develop an engineering component used in automobiles. In such engineering practices, it may not be wise to rely solely on the material properties of a natural fiber (the difference between the material properties of jute yarn and fibers is discussed in Section 5). Since bundles of jute fibers (i.e., sections of jute yarn), and not a single fibers are the basis of jute-based products, the material properties of jute yarns are needed to make various design and manufacturing decisions. Thus, we need to understand the material properties of both jute fibers and yarns. Otherwise, the reliability of the design decision may be uncertain. Information regarding jute fibers can be extracted from the works of numerous authors (e.g., [28,29,30,31,32,33,34,35,36,37,38]). Information on jute yarn, however, is not available. Therefore, the following section reports on the results of tensile tests performed on jute yarn specimens.

## 3. Tensile Test of Jute Yarns

A tensile test allows determination of the following properties: tensile strength, modulus of elasticity, strain to failure, and ductility (energy absorption pattern leading to failure). This section reports the results of the tensile tests performed on some specimens of jute yarn.

**Figure 2:**
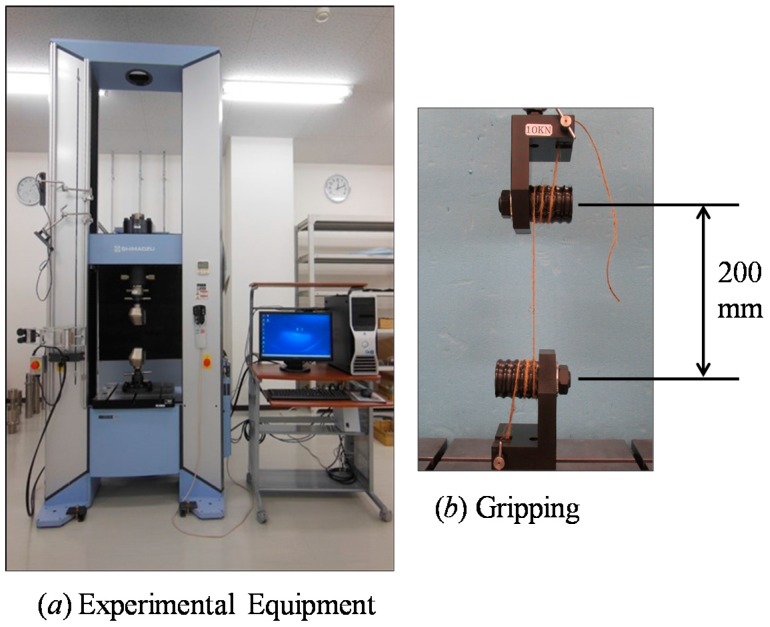
Experimental setup for tensile testing of jute yarn.

**Figure 3:**
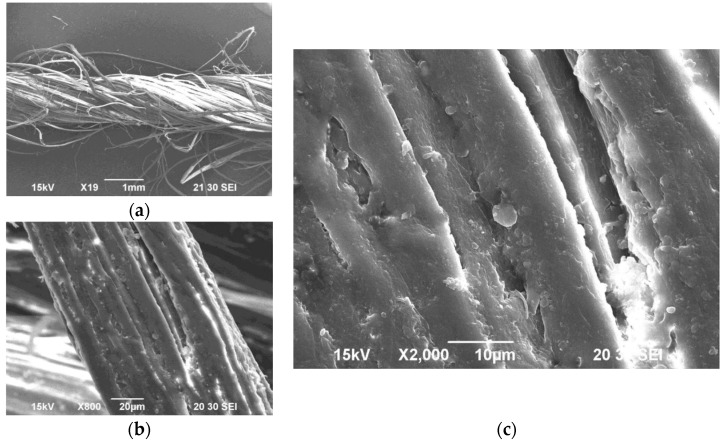
Magnified View of a jute yarn specimen. (**a**) A section of yarn; (**b**) A fiber in the yarn; (**c**) The surface of a fiber in the yarn.

Figure 2 shows the experimental setup used in this study. The jute yarns used in this study were collected from the Bangladesh Jute Research Institute (BJRI, http://www.bjri.gov.bd/) located in Dhaka (the capital of Bangladesh). The yarn count was 10^S^ (single ply) with a diameter of 1.7 mm (see Figure 1). The diameter was measured by an ordinary micrometer. The value of the diameter shown above was the average value of several trials (rounded to one decimal place). A universal tensile testing machine (Autograph AG-X; make: Shimadzu Corporation, Kyoto, Japan) was used to perform the tensile tests, as shown in Figure 2. A set of 15 specimens was prepared, each having a length of approximately 1.1 m. The grip-to-grip length of a specimen was fixed at 200 mm in the tensile tests, as shown in Figure 2. To avoid deflection, a preload of 5 N was applied to the specimen. The elongation velocity was 100 mm/min in all tests. Figure 3 shows a magnified image (SEM image) of the surface of a yarn specimen used in this study.

**Figure 4:**
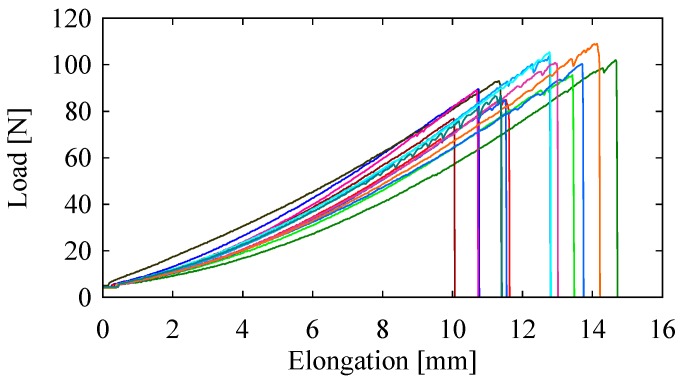
Load versus elongation plots of jute yarn specimens.

Figure 4 shows plots of load versus elongation for the fifteen jute yarn specimens. As seen from the plots in Figure 4, the yarns failed at an elongation of approximately 10–15 mm, and a load of approximately 75–115 N. Using the results shown in Figure 4, the tensile strength, modulus of elasticity, and strain to failure of each specimen were calculated. In particular, the maximum load (the load just before the failure) was divided by the cross-sectional area of the yarn to calculate the tensile strength. The elongation just before the failure was divided by the initial length (200 mm) to calculate the strain to failure. The tensile strength was divided by the strain to failure to calculate the modulus of elasticity. The results are summarized in Table 1.

The uncertainties underlying the tensile strength, modulus of elasticity, and strain to failure are described in Section 5.

## 4. Energy Absorption Characteristics

In addition to calculations of the tensile strength, modulus of elasticity, and strain to failure for jute yarn, the load versus elongation data shown in Figure 4 can provide additional insights into the failure mechanisms of jute yarns. Hence, we considered the issue of energy absorption. For a better understanding, let *F_i_* and *l_i_* be the load and instantaneous length of the jute yarn specimen in the *i*-th instance (*i* = 0, 1, 2...), respectively. Let *F_i_*_+1_ and *l_i_*_+1_ be the load and instantaneous length of the jute yarn specimen in the next instance (i.e., (*i* + 1)-sh instance), respectively. The instantaneous energy absorbed by a jute yarn specimen, denoted by *E_i_*, is given as follows:

(1) $$E_{i}=\left(\frac{F_{i}+F_{i+1}}{2}\right)\left(l_{i+1}-l_{i}\right)$$

**Figure 5:**
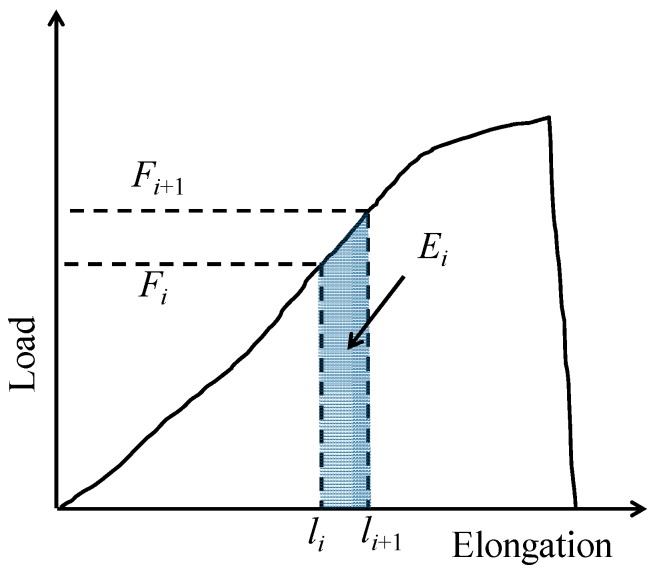
The concept of energy absorption.

Figure 5 schematically illustrates *E_i_*. As *F_i_* gradually increases with *l_i_* (see Figure 4), *E_i_* also increases with *l_i_*. The degree of increment may vary from one instance to another.

The variability in *E_i_* can be used as an indication of failure. Thus, we introduced an additional parameter called energy difference (denoted by *G_i_*), as follows:

(2) $$G_{i}=E_{i+1}-E_{i}$$

The energy difference *G_i_* can be observed with respect to the instantaneous true strain *ε_i_*, where the instantaneous true strain is given as

(3) $$\varepsilon_{i}=\ln\left(\frac{l_{i}}{l_{0}}\right)$$

**Figure 6:**
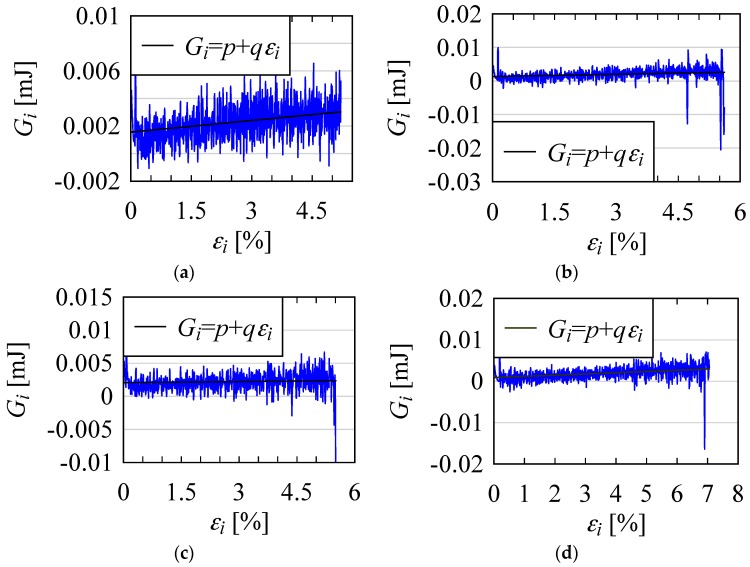
Energy difference patterns of four jute yarn specimens. (**a**) *p* = 0.001562, *q* = 0.0002815; (**b**) *p* = 0.001332, *q* = 0.0002262; (**c**) *p* = 0.00207, *q* = 0.00005224; (**d**) *p* = 0.0008807, *q* = 0.000292.

Note that in this particular case, *l*_0_ = 200 mm (see Figure 2). To see how *G_i_* varied with *ε_i_*, the plots of *G_i_* versus *ε_i_* were constructed. For example, Figure 6 shows four of these plots. The trend lines are also shown in the respective plots.

**Figure 7:**
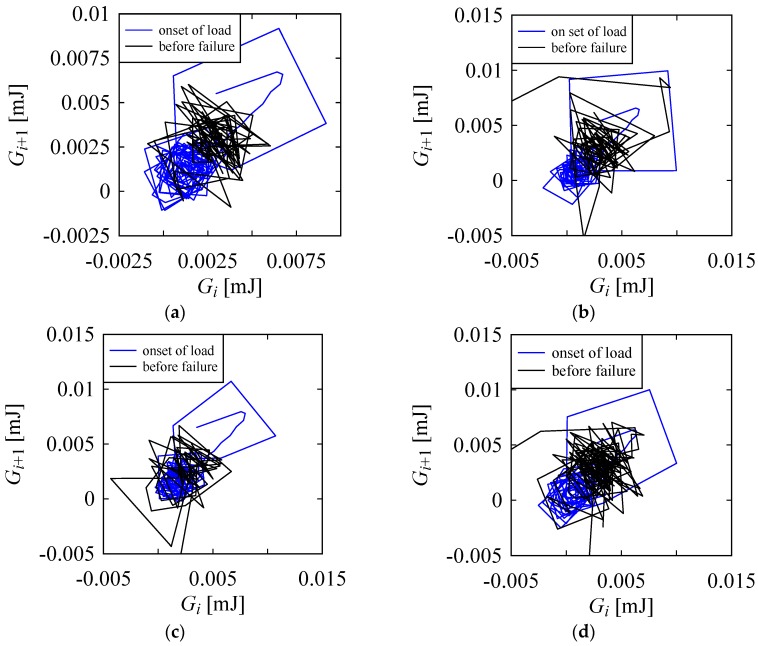
Return maps of energy difference at the onset of load and before failure. (**a**) Specimen 1; (**b**) Specimen 2; (**c**) Specimen 3; (**d**) Specimen 4.

As seen from Figure 6, for all four cases, *G_i_* exhibited a positive trend (i.e., the slopes of the trend lines given by *q* as shown in Figure 6 were all positive). This means that although *G_i_* fluctuated significantly, it gradually increased with increasing *ε_i_*. To visualize this energy difference characteristic, the return maps (i.e., the plots of the points (*G_i_*, *G_i_*_+1_), *i* = 0, 1, 2...) were constructed, as shown in Figure 7. It is worth mentioning that a return map (or a delay map) is often used to study a non-linear phenomenon (see [39] for more details). Notice the relative positions of the return maps in Figure 7. The return map of the energy difference at the onset of load was placed at a lower position compared to its position before failure. This was true for all four plots. The variability in *G_i_* before failure was very high. The argument held for the onset of load. This means that when a significant fluctuation in the energy absorption is observed after a certain amount of deformation, the yarn will soon fail.

**Figure 8:**
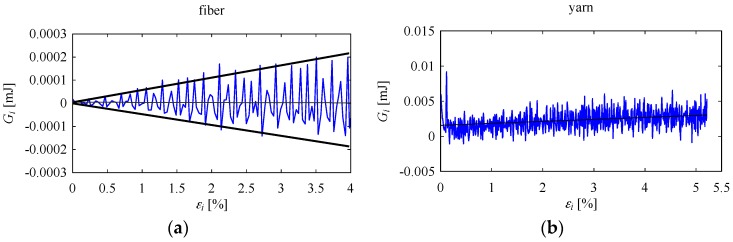
Comparison between (**a**) jute fiber and (**b**) jute yarn (copy of Figure 6a) in terms of energy difference.

Both jute yarns and jute fibers exhibited somewhat similar energy difference patterns. For example, consider the case shown in Figure 8, which plots energy difference for a jute fiber. Instability in the energy difference gradually increased with increasing true strain, producing an energy difference plot with a funnel-like shape. The trend in energy difference was horizontal (i.e., it did not increase or decrease, but fluctuated centered at *G_i_* = 0). In Figure 8b (a copy of Figure 6a), an energy difference plot for a jute yarn is shown for comparison. As seen in Figure 8, although the jute fiber exhibited a somewhat different instability pattern than the jute yarn, high instability was observed before failure for both cases. This implies that after a certain amount of true strain, both fiber and yarn fail because they cannot withstand the energy difference instability. In other words, the cause of failure in jute fiber or yarn is energy difference instability. Other natural materials may exhibit similar behavior. However, this remains an open topic for future research.

To make a jute yarn or fiber sufficiently strong, the energy difference variability must be controlled. This means that those who study different methods for improving the mechanical properties of jute (or other natural materials) can consult energy difference plots (along with information on other material properties) to elucidate the effectiveness of their methods.

## 5. Uncertainty Quantification

As mentioned earlier, a degree of uncertainty is associated with material properties. This uncertainty is even more prevalent for the material properties of natural materials. Therefore, uncertainty quantification is an important aspect of studying natural material properties. Without uncertainty quantification, natural material based product development processes may not provide the desired outcome. Numerous studies have tried to quantify the uncertainty exhibited by the material properties of natural materials using a probability distribution called a Weibull distribution. For example, Silva et al. [29] quantified the variability in material properties of sisal fibers using a Weibull distribution and correlated sisal microstructures with tensile strength. Defoirdt et al. [33] used a Weibull distribution to explain variability in the tensile properties of jute, bamboo, and coconut fibers. Fidelis et al. [9] examined the morphology of the natural fibers, and correlated their mechanical properties with their morphology using a Weibull distribution. Hossain et al. [38] created a histogram for the morphological structures of jute fiber cross-sectional areas, and provided ranges for their material properties. In all of these studies [9,29,33,38], significant variability was observed for the respective material properties studied. The remainder of this section uses both statistical and logical analyses to quantify variability in the tensile properties of jute yarn. A comparison with the jute fibers is also presented.

### 5.1. Statistical Approach

Let *TS*, *E*, and *s* be the tensile strength, modulus of elasticity, and strain to failure of a material. Let *x* be a variable so that *x* ∈ {*TS*, *E*, *s*}. In addition, let *x*(*i*) ∈ ℜ, *i* = 1, ..., *m* be *m* the number of experimental data points of *x*. The average ($\bar{x}$) and standard deviation (*sd*) of the data points are as follows:

(4) $$\bar{x}=\frac{\sum_{i=1}^{m}x\left(i\right)}{m}\;,\;\;\;\;\;\;\;\;\;\;\;sd^{2}=\frac{\sum_{i=1}^{m}{\left(x\left(i\right)-\bar{x}\right)}^{2}}{m-1}$$

We can estimate the uncertainties associated with the expected value of *x* and with its variance denoted as *μ* and *σ*^2^, respectively, using some standard statistical procedures. One of the most widely used procedures is to estimate the ranges *μ* and *σ*^2^, as follows:

(5) $$\mu \in \;\left[\bar{x}\pm t_{1-\alpha /2,\nu }\frac{sd}{\sqrt{m}}\right]\;,\;\;\;\;\;\;\sigma^{2}\in \left[\frac{v\times sd^{2}}{\chi_{1-\alpha /2,\nu }^{2}},\frac{v\times sd^{2}}{\chi_{\alpha /2,\nu }^{2}}\right]$$

In Equation (5), *ν* is the degrees of freedom equal to *m* − 1, *α* is the significance level, $t_{1-\alpha /2,\nu }$ is the critical value of a Student’s *t*-distribution for a two-sided test, $\;\chi_{\alpha /2,\nu }^{2}$ and $\chi_{1-\alpha /2,\nu }^{2}$ are the lower and upper critical values of a *χ*^2^ distribution for a two-sided test. Refer to [40,41,42] for the details of the relevant distributions. Note that for a two-sided test, (1 − *α*/2) × 100% is the confidence interval. Usually, we set an *α* = 0.05% or *α* = 97.5% confidence interval to calculate the ranges of the expected values and variance or standard deviation [the square root of variance $\sigma^{2}\;$ defined in Equation (5) is the standard deviation ($\sqrt{\sigma^{2}}$) that should not be confused with *sd* defined in Equation (4) from a given set of data points. Table 2 summarizes the ranges of the expected values and standard deviations of *TS*, *E*, and *s* calculated using the data points shown in Table 1. The minimum, maximum, average, and standard deviation (*sd*) of *TS*, *E*, and *s* are also shown in Table 2.

Consider the case of *TS*. The upper expected value of *TS* is 45.60 MPa + 6.32 MPa = 51.92 MPa, which is quite high, given the maximum value of *TS* (48.04 MPa). The lower expected value of *TS* is 37.76 MPa − 2.94 MPa = 34.82 MPa, which is higher than the minimum value of *TS* (33.87 MPa). In addition, consider the case of *E*. The upper expected value of *E* is 0.72 GPa + 0.06 GPa = 0.78 GPa, which is quite high given the maximum value of *E* (0.73 GPa). The lower expected value of *E* is 0.64 GPa − 0.03 GPa = 0.61 GPa, which is equal to the minimum value of *E* (0.61 GPa). Finally, consider the case of *s*. The upper expected value of *s* is 6.87% + 1.09% = 7.96%, which is quite high given the maximum value of *s* (7.4%). The lower expected value of *s* is 5.53% − 0.51% = 5.02%, which lower than the minimum value of *s* (5.03%). If we increase the confidence interval and recalculate the ranges of expected values and variances of the respective material properties, then the ranges of the expected values for all properties become wider compared to those observed in the data points (Table 1). On the other hand, if we decrease the confidence interval, then the ranges of the expected values of the respective material properties become narrow compared to those observed in the data points (Table 1). This means that when we try to quantify the uncertainty of a material property using an expected value that is estimated from a limited number of data points, there is a high possibility that we have a very strict or loose estimation, depending on the confidence interval.

Even if we avoid the above statistical analyses, and use a more straightforward approach, we may obtain similar results. For example, consider the following approach: uncertainty associated with *x* is estimated by $\bar{x}\pm sd$ as defined in Equation (4). Many authors have adopted this approach, as we observe in the literature cited in this article. Based on this approach, the uncertainty associated with *TS* is a range [37.67, 44.69] MPa, a highly truncated range compared to the range derived from its minimum and maximum values, i.e., [33.87, 48.04] MPa. Similarly, the uncertainty associated with *E* is a range [0.64, 0.72] GPa that is highly truncated compared to the range derived from its minimum and maximum values, i.e., [0.61, 0.73] GPa. In addition, the uncertainty associated with *s* is a range [5.51%, 6.89%] that is highly truncated compared to the range derived from its minimum and maximum values, i.e., [5.03%, 7.4%].

In synopsis, statistical analyses estimate both highly pessimistic and optimistic values of *TS*, *E*, and *s* of jute yarns, which may or may not be included in the experimental data points.

### 5.2. A Possibilistic Approach

**Figure 9:**
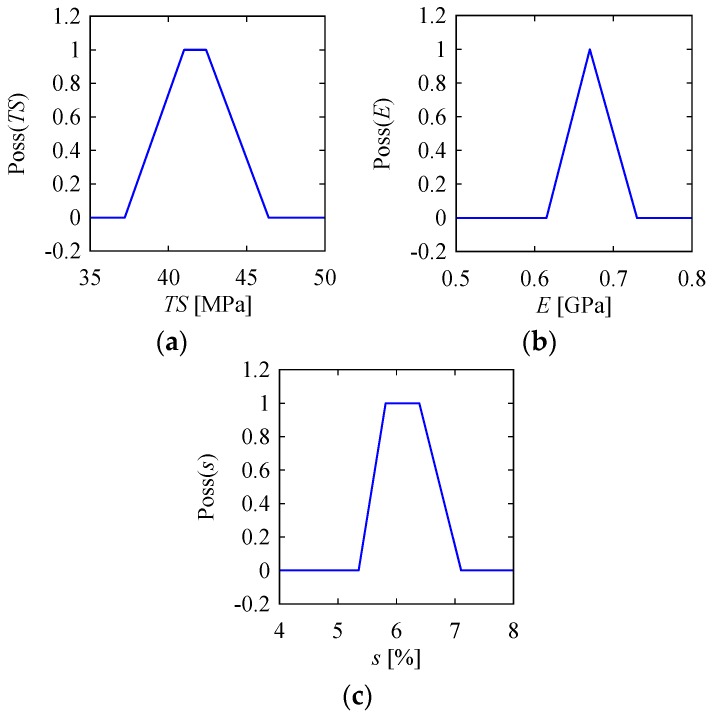
Possibility distributes of (**a**) tensile strength; (**b**) modulus of elasticity; and (**c**) strain to failure of jute yarns.

Apart from the statistical approaches to quantify the uncertainty associated with a material property, we considered other alternatives. Since the number of data points was small (15 data points), it was difficult to deduce a probability distribution (i.e., Weibull distribution) considering that the underlying uncertainty was the result of the small sample size. In fact, when it is unknown which distribution should be used to quantify the uncertainty, or even when there is insufficient data to deduce a probability distribution, the answer is to use a probability distribution-neutral representation of uncertainty. This was quite relevant for the case in this study, because of the limited number of data points (15 data points for each property, as shown in Table 1). As such, the concept of possibility [43] or possibility distribution [44] could be used. A possibility distribution is popularly referred to as a fuzzy number (see [2] for a definition). A possibility distribution entails a family of probability distributions (e.g., a triangular fuzzy number can entail a set of unimodal probability distributions, e.g., normal distribution, triangular distribution, and uniform distribution, [45,46]). In addition, a possibility distribution can also be deduced from a limited number of data points [45,46]. Such a distribution also provides a reliable representation of the uncertainty, which is compatible with the general concept of uncertainty [47]. For these reasons, we used possibility distributions instead of probability distributions to quantify the uncertainty associated the material properties shown in Table 1. In particular, we used a probability-possibility transformation as defined in [45] to deduce three fuzzy numbers (or possibility distributions) representing the uncertainty associated with *TS*, *E*, and *s*. Figure 9 shows the possibility distributions deduced from the data points shown in Table 1.

The deduced membership functions of *TS*, *E*, and *s* for jute yarn denoted by Poss(*TS*), Poss(*E*), and Poss(*s*), respectively, are as follows:

(6) $$\mathrm{Poss}\left(TS\right)=\max\left(0,\min\left(1,\frac{TS-37.2}{41-37.2},\frac{46.4-TS}{46.4-42.4}\right)\right)$$

(7) $$\mathrm{Poss}\left(E\right)=\max\left(0,\min\left(\frac{E-0.615}{0.67-0.615},\frac{0.73-E}{0.73-0.67}\right)\right)$$

(8) $$\mathrm{Poss}\left(s\right)=\max\left(0,\min\left(1,\frac{s-3.355}{5.815-3.355},\frac{7.105-s}{7.105-6.39}\right)\right)$$

Poss(*TS*) takes the shape of a triangular fuzzy number, whereas the other two take the shape of trapezoidal fuzzy numbers. Using the membership functions defined in Equations (6)–(8), the uncertainty associated with the *TS*, *E*, and *s* of jute yarn could be calculated. In this case, we used an operation called alpha cut [2], i.e., the range corresponding to Poss(.) = alpha ∈ [0,1]. Some of the important alpha cuts are summarized in Table 3. The alpha cut at Poss(.) = 0 corresponds to the largest range that is called the support of a possibility distribution. Thus, the supports of *TS*, *E*, and *s* were [37.2, 46.4] MPa, [0.615, 0.73] GPa, and [3.355%, 7.105%], respectively. Note that a support does not contain the minimum and maximum values of the data points. This means that the deduced possibility distribution considers the extreme data points (minimum and maximum values) as outliers and excludes them from the uncertainty quantification. Therefore, minimum and maximum values are automatically truncated. The most possible value(s) correspond(s) to Poss(.) = 1, which is a point for *E* and are two ranges for the other two properties, as shown in Table 3. The ranges corresponding to Poss(.) = 0.5 are the logically consistent ranges. This means that any propositions made taking a range Poss(.) > 0.5 are true more than they are false. For example, consider the following proposition: “*TS* of jute yarn is 39.5 MPa”. The truth-value of this proposition is 0.605263158 = Poss(*TS* = 39.5) [refer to Equation (6)]. This means it is somewhat true that the *TS* of jute yarn is 39.5 MPa. Therefore, this value of *TS* can be considered while designing a product using jute yarns. Consider another proposition: “The *TS* of jute yarn is 38 MPa”. The truth-value of this proposition is 0.210526316 = Poss(*TS* = 38). This means that it is somewhat false that the *TS* of jute yarn is 38 MPa. Therefore, this value of *TS* can be avoided, when designing a product using jute yarns. In synopsis, when we need a design range of a material property of jute yarns, we may take the range corresponding to Poss(.) = 0.5, because the points included in this range are true more often than they are false.

In addition, a possibility distribution provides the expected value or mean of the underlying parameter. In this case, we used the centroid method [2]. Table 3 also shows the centroid method-based expected values of *TS*, *E*, and *s* calculated from the respective possibility distributions. When no other details are given or available, we can consider these expected values for design and manufacturing decisions. Note that an expected value may or may not be a point in the core of the possibility distribution. Here, a core means the range or point corresponding to Poss(.) = 1 (i.e., the most possible value(s)). The expected value of *s* is not included in its core. This is not the case for the other two properties.

**Figure 10:**
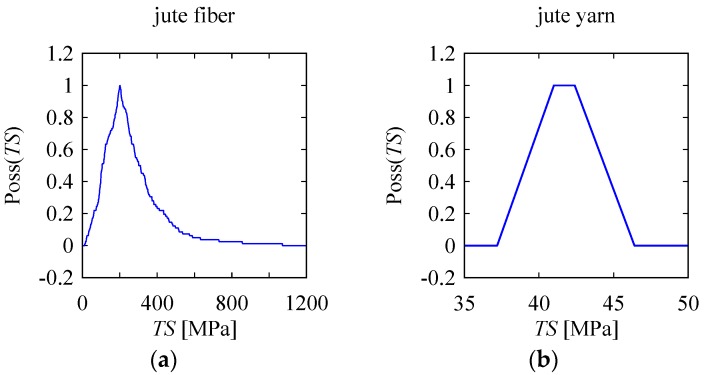
Comparison between jute fiber and yarn in terms of uncertainty in TS. (**a**) Fiber [10]; (**b**) Yarn (copied from Figure 9a).

It is worth mentioning that when we considered the *TS* and *E* values of jute fibers (see [10,33,34,35,36,37,38]), their values were too high in comparison to those given in Table 1. For a better understanding, the possibility distributions of jute yarn and jute fibers are shown side-by-side in Figure 10. The possibility distribution for the *TS* of jute fibers shown in Figure 10, taken from [10], was determined by the same methodology as that described in [44].

As seen from the *TS* possibility distribution for jute fibers shown in Figure 10, the degree of the associated uncertainty was very high. This was not the case for the possibility distribution of *TS* for jute yarn, also shown in Figure 10. This means that combined strength of some very strong and weak jute fibers create the resultant strength of the jute yarn. Since a bundle of jute fibers (not a single fiber) supports the strength of a product, the material properties of jute yarn, as summarized in Table 2 and Table 3, can be used for making various design decisions.

## 6. Concluding Remarks

(1) This study clearly identified the tensile strength, modulus of elasticity, and strain to failure of jute yarns. This study also quantified the uncertainty associated with these properties, using both statistical analyses and possibility distributions. From a comparison of the results obtained here with those of the jute fibers, the results of jute fibers exhibited very high values and uncertainties. Therefore, we recommend users to employ the results for jute yarn properties for making design decisions, while developing a jute-based product.
(2) To understand the failure mechanisms of both jute yarns and jute fibers, we considered the issue of energy absorption. Instability in the differences in energy absorption from one instance to another was observed for both jute yarns and fibers. Although the jute fiber exhibited a somewhat different instability pattern compared to the jute yarn, in both cases, high instability in energy absorption was observed before failure. This implies that after a certain amount of true strain, neither the fiber nor the yarn can withstand instability in energy absorption differences. At this point, failure occurs. Other natural materials may exhibit similar behavior. This remains an open issue for future research.

## Figures and Tables

**Table 1:** Properties of jute yarn.

Specimen No.	Tensile Strength (MPa)	Modulus of Elasticity (GPa)	Strain to Failure (%)
1	42.65	0.68	6.27
2	38.24	0.67	5.71
3	48.04	0.68	7.11
4	44.44	0.68	6.51
5	44.22	0.64	6.88
6	46.43	0.72	6.41
7	45.70	0.71	6.40
8	37.39	0.65	5.78
9	39.20	0.73	5.37
10	33.87	0.67	5.03
11	42.13	0.62	6.74
12	44.94	0.61	7.37
13	41.00	0.72	5.70
14	37.46	0.64	5.82
15	39.48	0.73	5.38

**Table 2:** Statistical uncertainties.

Parameters		Properties (*x*)		
		*TS* (MPa)	*E* (GPa)	*s* (%)
Minimum		33.87	0.61	5.03
Maximum		48.04	0.73	7.37
Average ($\bar{x}$)		41.68	0.68	6.17
Standard Deviation (*sd*)		4.01	0.04	0.69
Confidence Interval 95%	Expected Value (*μ*)	[37.76, 45.60]	[0.64, 0.72]	[5.53, 6.87]
	Standard Deviation ($\sqrt{\sigma^{2}}$)	[2.94, 6.32]	[0.03, 0.06]	[0.51, 1.09]

**Table 3:** Possibilistic uncertainties.

*Alpha*	*TS* (MPa)	*E* (GPa)	*s* (%)
0	[37.2, 46.4]	[0.615, 0.73]	[3.355, 7.105]
0.5	[39.1, 44.4]	[0.6425, 0.7]	[5.585, 6.7475]
1	[37.2, 42.4]	0.67	[5.815, 6.39]
Expected values (centroid method)	41.75	0.67	5.67
